# In-ovo feeding with creatine monohydrate: implications for chicken energy reserves and breast muscle development during the pre-post hatching period

**DOI:** 10.3389/fphys.2023.1296342

**Published:** 2023-12-14

**Authors:** Jonathan Dayan, Tal Melkman-Zehavi, Noam Goldman, Francesca Soglia, Marco Zampiga, Massimiliano Petracci, Federico Sirri, Ulrike Braun, Vivienne Inhuber, Orna Halevy, Zehava Uni

**Affiliations:** ^1^ Department of Animal Science, The Robert H. Smith Faculty of Agriculture, Food, and Environment, The Hebrew University of Jerusalem, Rehovot, Israel; ^2^ Koret School of Veterinary Medicine, The Robert H. Smith Faculty of Agriculture, Food, and Environment, The Hebrew University of Jerusalem, Rehovot, Israel; ^3^ Department of Agricultural and Food Sciences, Alma Mater Studiorum—University of Bologna, Cesena, Italy; ^4^ Alzchem Trostberg GmbH, Trostberg, Germany

**Keywords:** in-ovo feeding, creatine, glycogen, breast muscle, myogenic genes, proliferation, differentiation, broiler chicken

## Abstract

The most dynamic period throughout the lifespan of broiler chickens is the pre-post-hatching period, entailing profound effects on their energy status, survival rate, body weight, and muscle growth. Given the significance of this pivotal period, we evaluated the effect of in-ovo feeding (IOF) with creatine monohydrate on late-term embryos’ and hatchlings’ energy reserves and post-hatch breast muscle development. The results demonstrate that IOF with creatine elevates the levels of high-energy-value molecules (creatine and glycogen) in the liver, breast muscle and yolk sac tissues 48 h post IOF, on embryonic day 19 (*p* < 0.03). Despite this evidence, using a novel automated image analysis tool on day 14 post-hatch, we found a significantly higher number of myofibers with lower diameter and area in the IOF creatine group compared to the control and IOF NaCl groups (*p* < 0.004). Gene expression analysis, at hatch, revealed that IOF creatine group had significantly higher expression levels of myogenin (MYOG) and insulin-like growth factor 1 (IGF1), related to differentiation of myogenic cells (*p* < 0.01), and lower expression of myogenic differentiation protein 1 (MyoD), related to their proliferation (*p* < 0.04). These results imply a possible effect of IOF with creatine on breast muscle development through differential expression of genes involved in myogenic proliferation and differentiation. The findings provide valuable insights into the potential of pre-hatch enrichment with creatine in modulating post-hatch muscle growth and development.

## 1 Introduction

Meat-type chickens (broilers) have undergone intense selective breeding since the 1950s, aiming to achieve higher growth rates, better feed conversion, and increased meat yield. Consequently, modern broilers reach marketing weight in just 35–42 days (Havenstein et al., 2003a; Havenstein et al., 2003b; Zuidhof et al., 2014; Givisiez et al., 2020). This means that the 21-day of embryonic development (i.e., incubation period) and the early days post-hatch represent about 50% of the entire lifespan of modern broilers, hence, being of great importance to the production of poultry.

Throughout the broiler’s lifespan, the pre-to post-hatching period constructs the most dynamic period transitioning from late-term embryo to hatchling and growing chick (Uni and Ferket, 2004; Moran, 2007; De Oliveira et al., 2008). During this period, the skeletal muscle system switches from the second wave of myogenesis, starting at embryonic day (E) 8 until hatch, to the third wave of myogenesis, starting at E17 and continuing through post-hatch growth (Chal and Pourquié, 2017; Halevy and Velleman, 2022). The second myogenic wave is sustained essentially by cell fusion and adding myonuclei from proliferating Pax7+ progenitors (Otto et al., 2006; White et al., 2010). This is achieved by various factors, among them are Myogenic determination factor 1 (MyoD), which has a key role in myogenic commitment and lineage of the muscle progenitors (Davis et al., 1987), and Myogenin (MYOG) that acts downstream to MyoD and is crucial for terminal differentiation which includes the fusion of myoblasts to myotubes (Hasty et al., 1993; Zammit, 2017). During the third myogenic wave, MyoD is also involved in maintaining the pool of adult muscle stem cells -the satellite cells-, a subset of Pax7+ progenitors (Relaix et al., 2004; Gros et al., 2005; Kassar-Duchossoy et al., 2005; Zammit et al., 2006), which are the only source of new myoblasts required for the third myogenic wave. Muscle growth during the third myogenic wave is attributed primarily to myofiber hypertrophy (increased diameter of existing fibers through protein deposition) and to the proliferation and differentiation of satellite cells that either fuse to existing fibers or synthesize new myotubes (Hartley et al., 1992; Gokhin et al., 2008; Sparrow and Schöck, 2009). Two key factors involved in myoblast proliferation, terminal differentiation, and muscle hypertrophy in broilers are Insulin-like growth factor 1 (IGF1) and MYOG (Halevy et al., 2001; Halevy et al., 2004).

In order to promote muscle growth of embryos and hatchlings, energy supply is attained through egg nutrients (yolk and albumen) and later through exogenous nutrition (Moran, 2007; Uni et al., 2012). The transition from embryo to hatchling includes a dramatic change in energy metabolism, as towards hatch, the embryo switches from chorioallantoic to pulmonary respiration (De Oliveira et al., 2008; Cogburn et al., 2018), and energy production switches from oxidation of yolk derived lipids to anaerobic catabolism, by glycogenolysis and by gluconeogenesis (Tazawa et al., 1983; Decuypere et al., 2001). By the time the embryo hatches, its energy reserves are exploited, as almost all glycogen stores are depleted (Uni and Ferket, 2004; Uni et al., 2005; De Oliveira et al., 2008; Yadgary and Uni, 2012; Dayan et al., 2023a). Consequently, gluconeogenesis becomes the dominant pathway for energy provision supplied primarily by breast muscle protein degradation for amino acids (Donaldson, 1995; Keirs et al., 2002). This highlights the significance of the first exogenic feeding to support muscle development and integrity (Noy and Sklan, 1999; Halevy et al., 2000; Halevy et al., 2003; Bigot et al., 2003; Uni and Ferket, 2004; Kornasio et al., 2011).

This study investigates whether the elevation of late-term embryos’ energy stores will provide a jumpstart for breast muscle development and growth. For this aim, the method of in-ovo feeding (IOF), by inserting 0.6 mL of 1.5% creatine monohydrate solution into the embryo’s amniotic fluid three to 4 days before hatch, was applied according to Uni and Ferket (2003). The potential of creatine is derived from its high energetic value, with the possibility of rapid ATP provision through the creatine/phosphocreatine system (Bessman and Carpenter, 1985; Wyss and Kaddurah-Daouk, 2000). In chickens, creatine was shown to play a major role in energy metabolism, pivotal for the energetic status of hatchlings and their post-hatch performance (Zhang et al., 2016; Zhao et al., 2017a; Zhao et al., 2017b; Melo et al., 2022; Dayan et al., 2023a; Firman et al., 2023). Here, we provide an inclusive view of creatine and glycogen dynamics during the pre-to post-hatching period and in response to IOF with creatine in three essential tissues, breast muscle, liver, and yolk sac (YS) tissue, which are involved in energy supply and demand. To evaluate the effects of energy enhancement on early post-hatch breast muscle development, a novel, high-precision image analysis tool for histological evaluation was applied. In addition, we explored a possible mechanism underlying how energy status may modulate muscle development through differential expression of four genes involved in myoblast proliferation and differentiation (IGF1, MYOG, MyoD, and proliferating cell nuclear antigen- PCNA).

## 2 Materials and methods

### 2.1 In-ovo feeding calibration test

**FIGURE 1:**
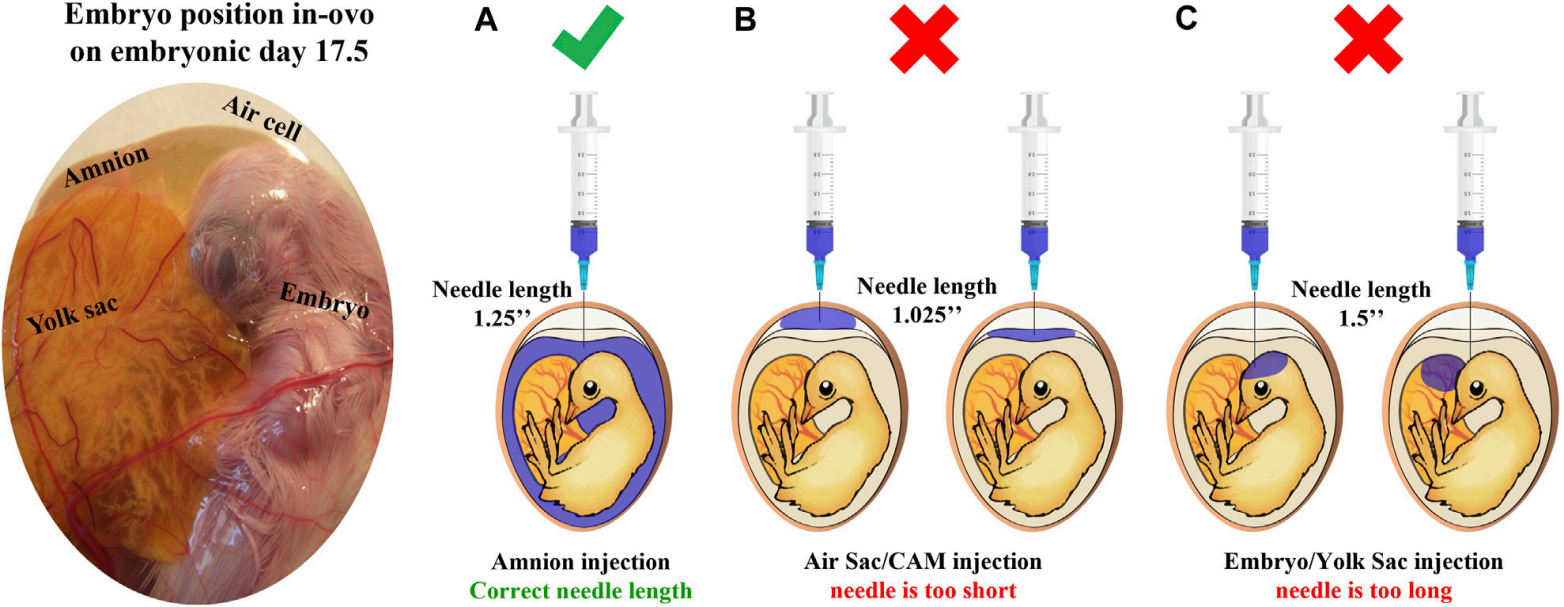
Site of injection test illustration. This test is designed to verify that the IOF solution reaches the amnion successfully. First, the amnion is identified by candling; next, needles in estimated lengths are chosen, and a sample of eggs is injected with 1 mL of coomassie blue dye (*n* = 20 eggs/examined needle). After cooling, eggs are opened to confirm successful injection with the correct needle. **(A)** A successful injection; only the amnion is stained. **(B)** An unsuccessful injection, the needle is too short, and the staining solution is found on top of the embryo reaching the air sac or chorioallantoic membrane. **(C)** An unsuccessful injection, the needle is too long, and the staining solution is found in embryonic tissues or yolk sacs.

Fertile eggs (*n* = 220; mean weight = 62.7 g, SD = 3.6 g) from 39-week-old broiler hens (Cobb 500) were purchased from a commercial breeder farm (Y. Brown and Sons Ltd., Hod Hasharon, Israel). Eggs were incubated in a Petersime hatchery at the Faculty of Agriculture of the Hebrew University under standard conditions (37.8°C and 56% relative humidity). On E10, eggs were candled, and unfertilized or dead embryo eggs were removed. Eggs were divided into two treatment groups: the control group (non-injected) and the IOF creatine group [injection volume of 0.6 mL with amount per embryo of 9 mg creatine monohydrate (Alzchem Trostberg GmbH, Germany) and 3 mg NaCl]. On E17.5, amniotic fluid (amnion) enrichment by in-ovo feeding (IOF) was performed according to the procedure developed by Uni and Ferket (2003). Eggs were injected according to treatments, and IOF was performed using a 21-gauge needle. The site of injection was verified in a pre-test designed to ensure that the IOF solution reached the amnion successfully (Figure 1). Once the IOF procedure was completed, all eggs were transferred to hatching trays, and hatchability was monitored. At hatch day, 24 male chicks per group were sampled and the rest were transferred to brooders at the Faculty of Agriculture of the Hebrew University and reared according to the breeder recommendations (Cobb-Vantress). During the 3-day rearing period, chicks were fed a standard commercial starter diet (formulated by Brown feed mill, Kaniel, Israel) with *ad-libitum* access to water and feed. Tissue sampling was performed on day of hatch and day 3 post-hatch. On each sampling day, 24 birds/treatment were randomly selected and euthanized by cervical dislocation, the body and breast muscle weights were recorded. Breast muscle (*Pectoralis major and Pectoralis minor*) percentage was calculated relative to body weight.

### 2.2 In-ovo feeding with creatine monohydrate-mode of action

Fertile eggs (*n* = 330; mean weight = 62.46 g, SD = 4.4 g) from 33-week-old broiler hens (Cobb 500) were purchased from a commercial breeder farm (Y. Brown and Sons Ltd., Hod Hasharon, Israel). Eggs’ incubation and handling were the same as described above. On E17.5, IOF was performed, and eggs were divided into three treatment groups: control group (non-injected), IOF NaCl group (injection volume: 0.6 mL with amount per embryo of 3 mg NaCl), and IOF creatine group [injection volume of 0.6 mL with amount per embryo of 9 mg creatine monohydrate (Alzchem Trostberg GmbH, Germany) and 3 mg NaCl]. After completion of the IOF procedure, all eggs were transferred to hatching trays, and hatchability was monitored. At hatch day, six male chicks per group were sampled and the rest were transferred to brooders at the Faculty of Agriculture of the Hebrew University at hatch and reared according to the breeder recommendations (Cobb-Vantress). During the 14-day rearing period, chicks were fed a standard commercial starter diet (formulated by Brown feed mill, Kaniel, Israel) with *ad-libitum* access to water and feed. Tissue sampling was performed on E17, E19, hatch day, day 1, day 3, day 6, and day 14. On each sampling day, six embryos/birds were randomly selected and euthanized by cervical dislocation. The following parameters were recorded: body weight, YS tissue weight (according to Yadgary et al., 2010), liver weight, and breast muscle weight. The relative weights of the liver, breast muscle, and YS tissue were calculated as a percent of body weight. Liver, YS tissue, and breast muscle (*Pectoralis major*) samples were collected to determine creatine and glycogen content (E17—day 1). In addition, breast muscle samples were collected for histology (day 14) and the evaluation of expression levels of myogenic genes (E17—day 6).

### 2.3 Analysis of creatine and glycogen energy resources

Tissue samples (approximately 500–1,500 mg per tissue) were collected, placed in liquid nitrogen, and kept at −80°C until further processing. Next, samples were lyophilized and examined for their creatine and glycogen content by Swiss-BioQuant-AG (Reinach, Switzerland). The concentrations of creatine and glycogen (mg/g dry weight tissue) were determined, and the total amount (mg) was calculated in order to demonstrate the full capacity of energy storage in each tissue as previously described (Yadgary and Uni, 2012; Dayan et al., 2023a).

### 2.4 Evaluation of muscle histomorphology

Muscle sampling for histology was performed as previously described by Halevy et al. (2004). Briefly, muscle samples (approximately 0.5 cm × 0.5 cm × 1 cm) were removed from the superficial region of the proximal half of the left *Pectoralis major* muscle. The muscle samples were fixed in 3.7% formaldehyde in PBS at pH 7.4 (Sigma-Aldrich, Rehovot, Israel) for 24 h. Then, samples were dehydrated, cleared, and embedded in paraffin. Cross sections of 4–6 µm thick were cut, deparaffinized in Histochoice clearing agent (Sigma-Aldrich St. Louis, MO), rehydrated, and stained with Picrosirius Red Fast Green staining to differentiate between myofibers (stained in green) and connective tissue (stained in red). After drying, samples were mounted on cover glass using DPX slide mounting medium (Sigma-Aldrich St. Louis, MO). Finally, images were captured using an EVOS FL Auto-inverted microscope. Images are comprised of 12 stitched fields of ×60 magnification.

The automated image analysis workflow was developed and performed as described by Dayan et al. (2023b). Briefly, the workflow for Fiji software is based on PT-BIOP, Cellpose wrapper, and MorphoLibJ plugin. Image processing included detecting myofibers and converting label masks to regions of interest (ROIs). The morphological analysis included extracting myofiber metrics from the ROIs; the lesser diameter (µm) and cross-sectional area (µm^2^) were measured, and the number of myofibers per mm^2^ was calculated.

### 2.5 Analysis of mRNA expression

Tissue samples were collected, placed in liquid nitrogen, and refrigerated at −80°C until further processing. Total RNA was isolated from 100 mg of tissue (YS tissue, liver, and breast muscle) using TRI-Reagent (Sigma-Aldrich, St. Louis, MO) according to the manufacturer’s protocol. RNA concentration was determined using a NanoDrop ND-1000 instrument (Thermo Fisher Scientific, Wilmington, DE). Total RNA was treated with DNase using a Turbo DNA-free Kit according to the manufacturer’s protocol (Ambion; Thermo Fisher Scientific, Wilmington, DE). cDNA was created from 1 µg of DNA-free RNA using the qPCRBIO cDNA synthesis kit according to the manufacturer’s protocol (PCRBIOSYSTEMS, London, United Kingdom). Relative mRNA expression was evaluated using gene-specific primers (Table 1): IGF1, involved in the induction of muscle cell differentiation and hypertrophy; MYOG, myogenic regulatory factor; MyoD, myogenic regulatory factor; PCNA, a marker for dividing cells, correlated with S phase in DNA replication; β-actin a housekeeping cytoskeletal protein, and HPRT, an enzyme in the purine synthesis in salvage pathway, also a housekeeping gene (Dayan et al., 2023a). Primer sequences were designed using Primer-BLAST software (Ye et al., 2012) based on published cDNA sequences purchased from Sigma-Aldrich (Rehovot, Israel). PCR products were validated by gel electrophoresis in 1.5% agarose gel. Real-time quantitative polymerase chain reactions (qPCR) were conducted in triplicate in a Roche Light cycler 96 (Roche Molecular Systems, Inc., Pleasanton, CA). Each reaction (20 µL) included 3 µL of cDNA sample diluted in ultrapure water in a ratio of 1:20 (UPW, Biological Industries, Beit HaEmek, Israel), 4 µM of each primer, and Platinum SYBR Green qPCR super mix-UDG (Thermo Fisher Scientific, Wilmington, DE). Reaction conditions were: preincubation at 95°C for 60 s followed by 40 cycles of a 2-step amplification cycle of 95°C for 10 s and 60°C for 30 s. The procedure was finalized with a melting curve generated in the following conditions: 95°C for 60 s, 65°C for 60 s, and 97°C for 1 s. Relative mRNA expression was calculated by subtracting the housekeeping gene’s geometric mean of cycle threshold (Ct) values from sample Ct (Pfaffl, 2007; Dayan et al., 2023a).

**TABLE 1:** In-ovo feeding calibration test.

Day	Treatment	Hatchability (%)	Body weight (g)	Breast muscle weight (g)	Breast muscle %
Hatch	Control	93.6 (N hatched = 88/94)	47.42 ± 0.55	1.81 ± 0.06	3.81 ± 0.10
	IOF Creatine	95.5 (N hatched = 86/90)	47.30 ± 0.63	1.99 ± 0.06*	4.21 ± 0.12*
Day 3	Control	—	87.85 ± 1.6	6.57 ± 0.21	7.48 ± 0.19
	IOF Creatine	—	82.99 ± 1.49	6.17 ± 0.17	7.43 ± 0.15

Comparison of hatchability (%), body weight (g), breast muscle weight (g), and breast muscle percent (relative to body weight) between control chicks and chicks that were in-ovo feeding (IOF) with creatine. Each IOF-treated embryo was injected with 0.6 mL of 1.5% creatine monohydrate (9 mg/embryo) dissolved in 0.5% NaCl (3 mg). Asterisk denotes means that are significantly different between treatments at each time point, as derived from Student’s t-test (*p* ≤ 0.05). For data of body weight, breast muscle weight, and breast muscle percent, *n* = 24 per treatment and day.

### 2.6 Statistical analysis

For the IOF calibration test, at each time point, differences between treatments were evaluated using Student’s *t*-test and considered significant with a *p*-value lower than or equal to 0.05 (*p* ≤ 0.05).

For the IOF “mode of action” test, differences between treatments at each time point were evaluated by one-way ANOVA analysis, followed by Tukey’s HSD test. Differences were considered significant with a *p*-value lower than or equal to 0.05 (*p* ≤ 0.05).

All values are presented as mean ± standard error mean (SEM). All statistical analyses were performed using JMP-pro 16 software (SAS Institute Inc., Cary, NC).

## 3 Results

### 3.1 In-ovo feeding calibration test

In order to examine the feasibility of creatine injection into broiler embryos, the site of injection and hatching performance test was performed. The IOF solution was designed based on solubility, osmolality (mmol/kg), and pH levels of the amniotic fluid (Omede et al., 2017). Our results prove that when correctly injected with a needle of appropriate length (Figure 1), IOF of creatine in a concentration of 1.5% will not affect embryonic mortality, as hatchability of the IOF creatine group was 95.5% compared to 93.6% of the non-injected control (Table 1). Furthermore, on the day of hatch, IOF creatine hatchlings had significantly higher breast muscle weight with a percentage of 4.2% compared to 3.8% in the control group (*p* < 0.02). On day 3 post-hatch, no significant differences were shown.

### 3.2 In-ovo feeding with creatine monohydrate-mode of action

Once optimal conditions were found, and a positive effect of IOF with creatine was observed on breast muscle weight and percentage, a “mode-of-action” experiment was done. In this experiment, the specific effects of creatine enrichment were examined on: (1) energy resources in three key tissues (breast muscle, liver, and YS tissue) and 2) breast muscle histological and molecular parameters during the critical days’ pre-to post-hatch [E17-D14]. To this purpose, three treatment groups were selected: control (non-injected), IOF creatine (injection volume: 0.6 mL; 9 mg/embryo of creatine monohydrate and 3 mg of NaCl), and IOF NaCl (injection volume: 0.6 mL; 3 mg/embryo of NaCl). As previously observed for the calibration test, hatchability was not affected by IOF as it ranged between 94.8% in the NaCl group to 95.04% in IOF creatine and 95.4% in the control group.

**FIGURE 2:**
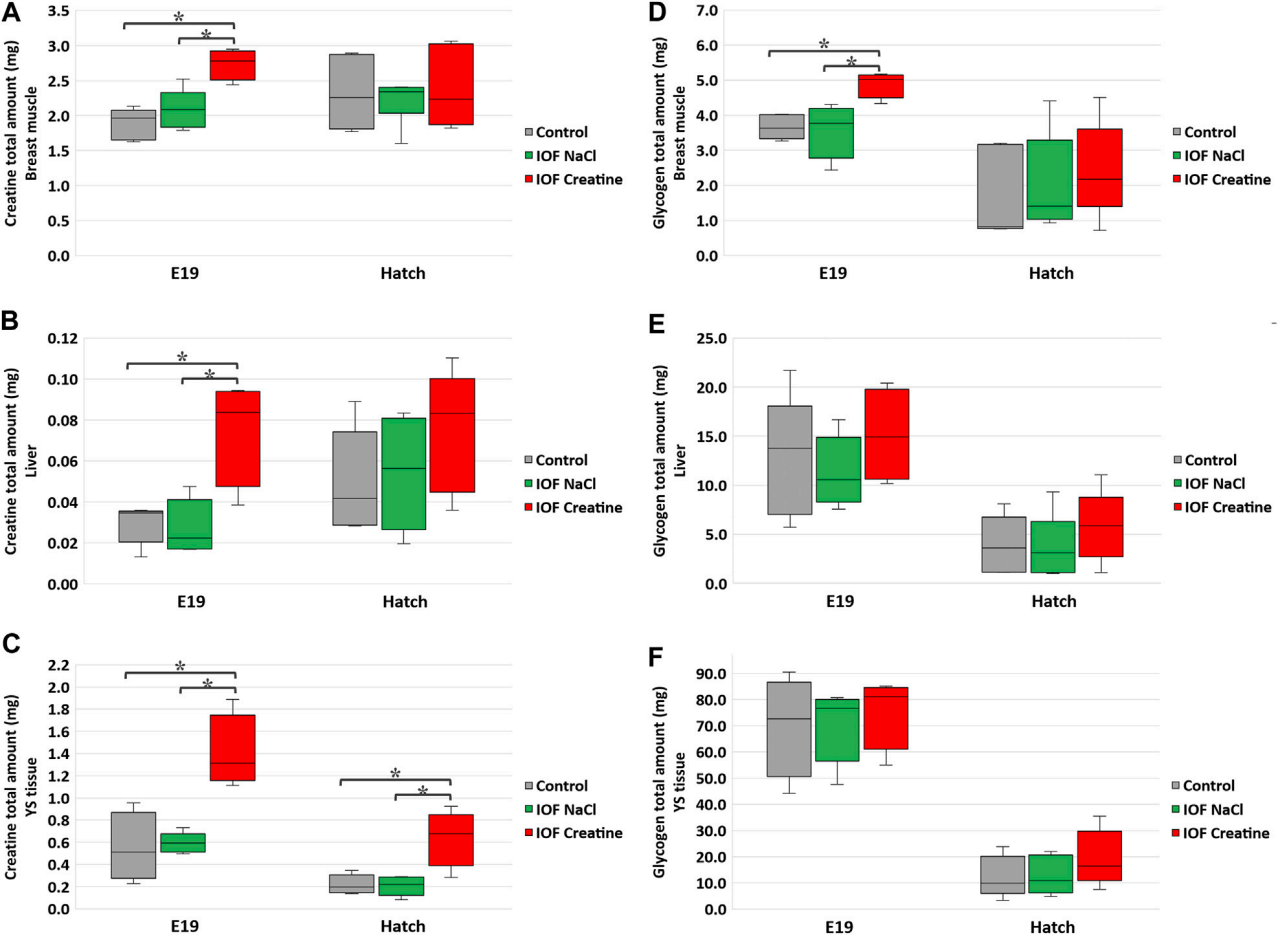
Creatine **(A–C)** and glycogen **(D–F)** levels per tissue on E19 and at hatch day of the Control, IOF NaCl, and IOF Creatine treatment groups. Levels are presented as total tissue creatine amount (mg) in breast muscle **(A, D)**, liver **(B, E)**, and YS tissue **(C, F)**. Asterisks denotes means that are significantly different between treatments at each time point, as derived from Tukey’s HSD test (*p* ≤ 0.05), *n* = 6 per treatment and day.

The total amount (mg) of creatine and glycogen were calculated in order to demonstrate the full capacity of energy storage in each examined tissue as previously described (Yadgary and Uni, 2012; Dayan et al., 2023a). Our results show that on E19, 48 h post-IOF, creatine levels were 45% higher, and glycogen levels were 30% higher (*p* < 0.03) in the breast muscle of the IOF creatine group compared to control and NaCl groups (Figures 2A, D). Analysis of liver and YS tissues on the same day revealed an even higher difference with more than 140% creatine amount (*p* < 0.01) compared to the control and NaCl groups (Figures 2B, C). As for liver and YS tissue glycogen, no significant differences were observed between treatments (Figures 2E, F). At hatch (Figure 2C), YS tissue creatine amount remained significantly higher in the IOF creatine group with over 180% difference (*p* < 0.004). Overall, between E19 and the day of hatch, in all treatments a significant reduction in glycogen amount in the breast muscle, liver, and YS tissue was observed (*p* < 0.01).

**FIGURE 3:**
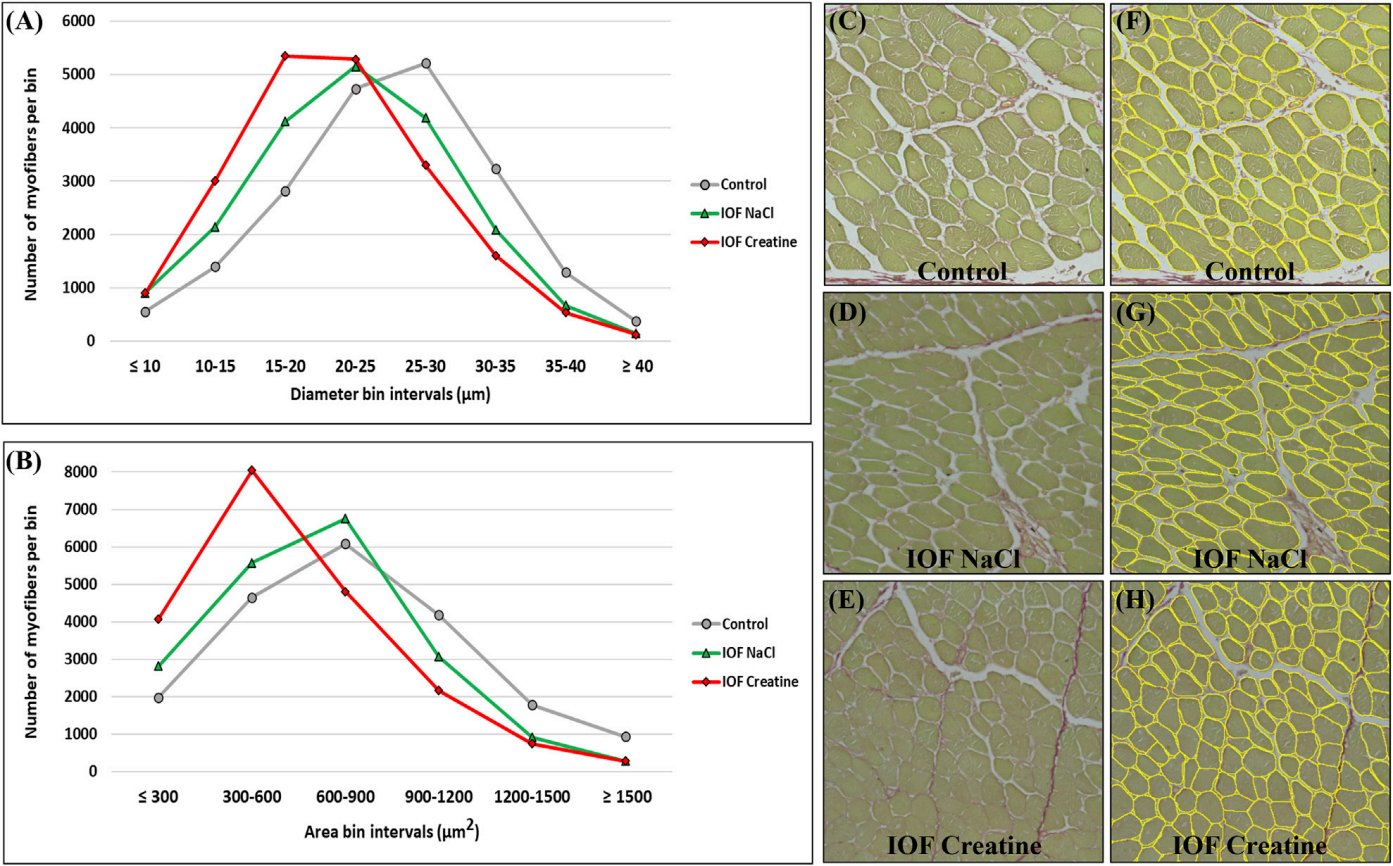
Myofiber distribution in the breast muscle (*pectoralis major*) of 14-day-old broiler. Myofibers are clustered in bin intervals where **(A)** displays the diameter distribution in intervals of 5 µm and **(B)** presents the area distribution in intervals of 300 µm^2^. **(C–H)** A representative field of original sirius red fast green stained images from the control **(C, F)**, IOF NaCl **(D, G)**, and IOF Creatine **(E, H)** treatments. Images were generated using an EVOS FL Auto-inverted microscope with ×60 magnification and are comprised of 12 stitched fields. Measurements were performed using Fiji ImageJ with Cellpose and MorphoLibJ plugins.

Breast muscle percentage was similar in all groups on day 14 post-hatch (16.32% ± 0.33%, 16.49% ± 0.31%, and 16.11% ± 0.79% in control, IOF NaCl, and IOF creatine groups, respectively). To gain further understating of these results we evaluated breast muscle myofibers’ histomorphology on day 14 post-hatch. To that end, a novel deep learning-based automated image analysis tool was applied according to Dayan et al. (2023b). Figures 3C–H shows representative images and the automated identification of myofibers. This enabled us to analyze tens of thousands of myofibers relatively quickly; 19,610, 19,415, and 20,103 for control, IOF NaCl, and IOF creatine treatments, respectively. Our results show that the average myofiber size of the IOF creatine group was significantly smaller (*p* < 0.0001) as the diameter and area were 21.4 and 577.4 µm^2^, respectively, compared to 22.4 µm, 667.1 µm^2^ of the IOF NaCl group and 24.9 µm, 787.2 µm^2^ of the control group (Table 2). Figures 3A, B presents the distribution of myofibers’ diameter and area, displaying a distinct Gaussian curve for the IOF creatine group with the highest rate of smaller myofibers. As expected, an opposing trend was demonstrated for the number of myofibers per mm^2^ (Table 2), with a significantly higher number of myofibers in the IOF creatine group compared to the control and the IOF NaCl groups (*p* < 0.004).

**TABLE 2:** Histomorphological analysis of the Control, IOF creatine, and IOF NaCl groups.

Treatment	Number of myofibers	Myofiber diameter (µm)	Myofiber area (µm^2^)	Number of myofibers per mm^2^
Control	19,610	24.97 ± 0.053^a^	787.21 ± 2.84^a^	1,268.52 ± 38.12^c^
IOF NaCl	19,415	22.43 ± 0.052^b^	667.13 ± 2.39^b^	1,494.34 ± 42.58^b^
IOF Creatine	20,103	21.14 ± 0.049^c^	577.38 ± 2.36^c^	1797.83 ± 105.38^a^

Summary of morphological analysis and comparison between the Control, IOF, creatine, and IOF, NaCl treatment groups in the pectoral muscle of a 14-day-old broiler. Myofiber diameter and area are presented as mean size ± standard error mean. The number of myofibers per mm^2^ was calculated for each image. The number of counted myofibers was normalized with the total myofiber area. Superscript letters denote the means that are significantly different between treatments, as derived from Tukey’s HSD, test (*p* ≤ 0.05), *n* = 84 (four birds per treatment and 7 images per bird). Images comprised of 12 stitched fields of ×60 magnification were generated using an EVOS FL, Auto-inverted microscope.

**FIGURE 4:**
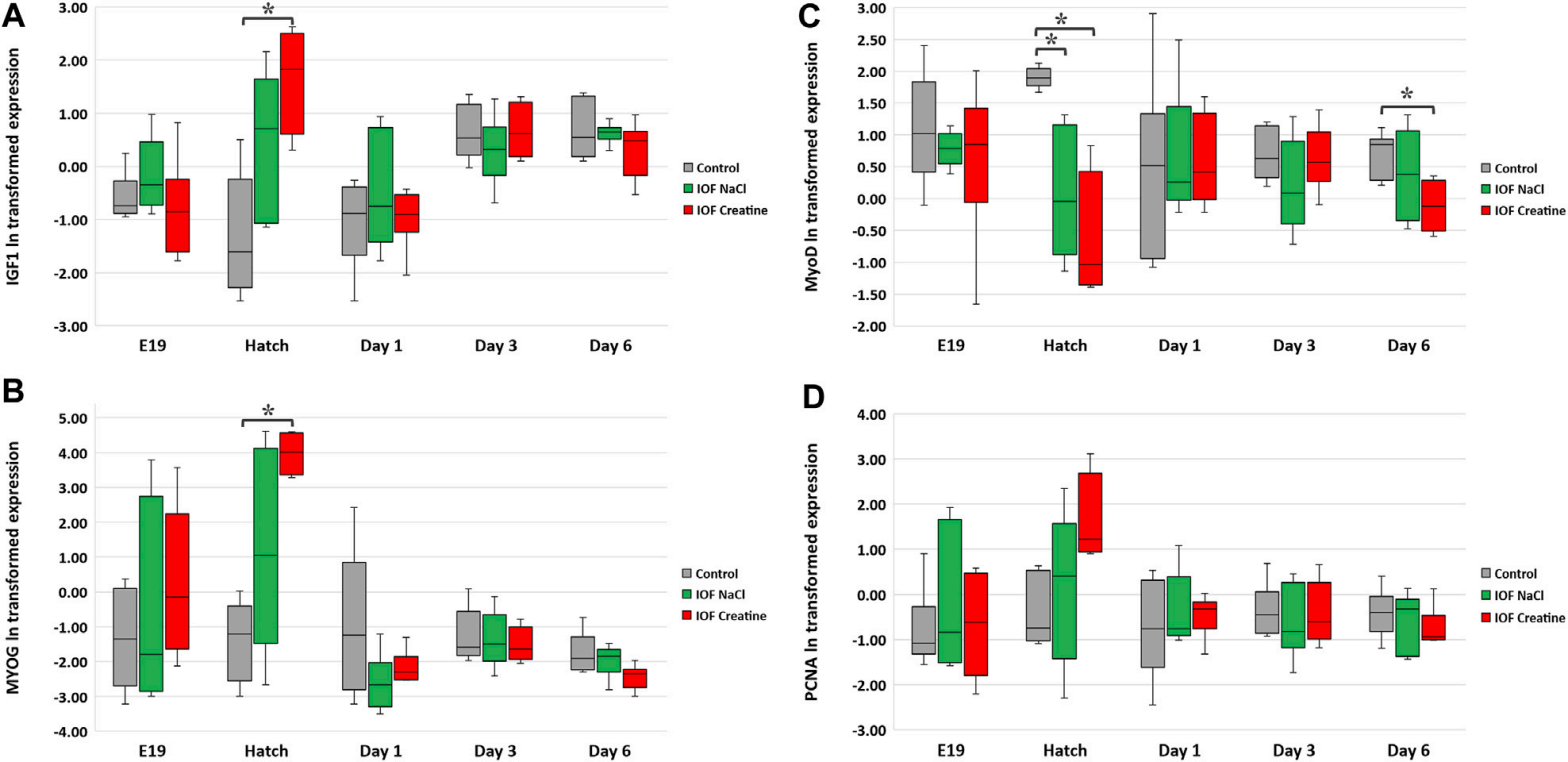
Expression analysis of breast muscle genes: **(A)** IGF1, **(B)** MYOG, **(C)** MyoD, and **(D)** PCNA. According to the heterogeneity of variance in embryonic exponential growth, logarithmic (ln) transformation was carried out for the data. Expression is normalized to GEOMEAN of HPRT, β-actin, and is relative to expression on E17 (before IOF). Asterisks denote means that are significantly different between treatments at each time point, as derived from Tukey’s HSD test (*p* ≤ 0.05), *n* = 6 per treatment and day.

At the molecular level, the evaluation of the “mode of action” on muscle development during the pre-to post-hatching period E17-D6 was performed by examining four genes involved in myoblast proliferation and differentiation; IGF1, MYOG, MyoD, and PCNA (Table 3). In order to evaluate the expression levels of these genes, in each timepoint, data were subjected to one-way ANOVA analysis followed by Tukey’s HSD test. For IGF1 and MYOG genes (Figures 4A, B), at hatch, significantly higher expression levels were found in the IOF creatine group, compared to the control (*p* < 0.01). For the expression of MyoD (Figure 4C), significant differences were found at hatch and on day 6, with higher expression levels in the control group compared to the IOF creatine group (*p* < 0.04). As for the expression of PCNA, although not significant, a trend for higher expression levels is shown in the IOF creatine group at hatch.

**TABLE 3:** Primers used for real-time PCR gene expression analysis.

Target^a^	Accession number	Primer F (5′-3′)	Primer R (5′-3′)	Amplicon size	References
IGF1	NM_001004384.2	GTA​TGT​GGA​GAC​AGA​GGC​TTC	TTGGCATATCAGTGTGGC	193	Kornasio et al. (2009)
MYOG	D90157.1	GCGGAGGCTGAAGAAGGT	AGGCGCTCGATGTACTGG	123	Chen et al. (2018)
MyoD	NM_204214.3	GAC​GGC​ATG​ATG​GAG​TAC​AG	GCT​TCA​GCT​GGA​GGC​AGT​A	200	Harding et al. (2016)
PCNA	NM_204170.3	GTGCAAAAGACGGTGTGA	ACC​TCA​GAG​CAA​AAG​TCA​GC	147	Primer Blast
HPRT	NM_204848	AAG​TGG​CCA​GTT​TGT​TGG​TC	GTA​GTC​GAG​GGC​GTA​TCC​AA	110	Boo et al. (2020)
β-actin	NM_205518.1	AAT​GGC​TCC​GGT​ATG​TGC​AA	GGC​CCA​TAC​CAA​CCA​TCA​CA	112	Primer Blast

^a^ Insulin-like growth factor 1 (IGF1), involved in the induction of muscle cell differentiation and hypertrophy; Myogenin (MYOG), myogenic regulatory factor; Myogenic differentiation protein 1 (MyoD), myogenic regulatory factor; Proliferating cell nuclear antigen (PCNA), a marker for dividing cells; hypoxanthine phosphoribosyl transferase 1 (HPRT) is an enzyme in the purine synthesis in salvage pathway and a housekeeping gene, and; β-actin, a housekeeping cytoskeletal protein.

## 4 Discussion

The pre-to post-hatching period is recognized as the most dynamic phase throughout the lifespan of broilers, with profound effects on their energy status, survival rate, body weight, post-hatch muscle growth, and the onset of growth-related myopathies (Christensen et al., 1999; Collin et al., 2007; Leksrisompong et al., 2007; Moran, 2007; De Oliveira et al., 2008; Kornasio et al., 2011; Oviedo-Rondón et al., 2020). Given the significance of this critical period, there is a growing interest in exploring the effectiveness of IOF as a potential strategy for optimizing the transition from late-term embryo to hatchling and growing chick (Oliveira et al., 2023). In addition, the selection of meat-type chicken lines for increased growth and muscle development has been accompanied by a significant decrease in muscle energy reserves (Berri et al., 2001). In this study, we evaluated the effect of the IOF with creatine on the energy levels of late-term embryos and hatchlings and post-hatch breast muscle development. We provide an inclusive view of the high-energy value molecules of creatine and glycogen simultaneously in the breast muscle, liver, and YS tissue. The results demonstrate that IOF with creatine elevates high-energy-value molecules in all tissues. On day 14 post-hatch, despite the evidence of highest energy levels post-IOF in the IOF creatine group, the mean myofiber diameter and area are lower than those found in the other groups. Gene expression analysis implies a possible effect of creatine enrichment by IOF on early post-hatch breast muscle development through differential expression of myogenic-related genes.

An ongoing controversy still exists about the exact method to perform IOF, sometimes leading to different results; clear descriptions are missing in many of the published studies where IOF was used (Oliveira et al., 2023). However, the present study provides a comprehensive view of the IOF procedure, including the site of the injection test designed to confirm the successful delivery of the injected solution to the embryo’s amniotic fluid (Uni and Ferket, 2003; Uni et al., 2005). Figure 1 illustrates the embryo’s position within the egg on E17.5 and outlines the verification process for optimal needle length. As for the timing of IOF, E17.5 marks the optimal time for injection due to the embryo’s favorable positioning, which facilitates relatively easy access to the amniotic fluid. Additionally, IOF at this stage ensures rapid delivery of the solution to the embryo’s intestine, as it coincides with the onset of rapid swallowing of the amniotic fluid (Romanoff, 1960). In terms of the effect on muscle development, E17.5 is just between the critical transition period from the second to the third wave of myogenesis (Stockdale, 1992), suggesting that this timing may offer an opportunity for modulatory effects on muscle development and growth (Kornasio et al., 2011). In addition, our study demonstrates the feasibility of creatine injection via IOF, and when performed correctly, IOF will not cause higher embryonic mortality. Indeed, hatchability was similar in all treatments. Moreover, the results revealed that IOF with creatine significantly increased breast muscle weight and percentage at day of hatch, corresponding with previous reports by Zhao et al. (2017a,b).

Creatine was shown to maintain an available energy supply in late-term embryos and hatchlings (Dayan et al., 2023a). This evidence and our present findings of higher creatine amount in the breast muscle, liver, and YS tissue in IOF creatine embryos and hatchlings, suggest their improved energy status. Moreover, in accordance with previous studies, it is suggested that the surplus of breast muscle’s creatine and glycogen in the IOF creatine group has slowed down breast muscle catabolism for energy provision towards hatch through protein mobilization and amino acid supply for the gluconeogenic pathway (Uni et al., 2005; Foye et al., 2006; De Oliveira et al., 2008; Kornasio et al., 2011). It can be concluded that the higher levels of creatine and glycogen have contributed to a higher breast muscle weight and percentage at hatch.

To further understand the similar values of breast muscle percentage found on day 14 post-hatch, the hypertrophy of breast myofibers has been evaluated by an automated image analysis tool (Dayan et al., 2023b). This tool enables us to analyze a large number of data sets, with maximal coverage for each examined cross-section, producing a better representation of the examined samples, while providing rapid and high-precision results. Distribution analysis of myofiber diameter and area exhibited the typical Gaussian curve, similar to that reported in previous publications (Halevy et al., 2006; Piestun et al., 2009; Piestun et al., 2011; Piestun et al., 2017; Patael et al., 2019). Surprisingly, despite the evidence of the highest energy levels on E19 and hatch, the IOF creatine group had significantly smaller myofibers with lower mean myofiber diameter and area on day 14. Our findings of decreased myofiber size following the treatment of IOF with creatine differ from those reported by Zhao et al. (2017b) and Firman et al. (2023). Zhao et al. (2017b) used creatine pyruvate, a different creatine source, which has a lower bioavailability compared to the creatine monohydrate used in our study (Deldicque et al., 2007a; Jäger et al., 2007). Firman et al. (2023) applied a different IOF protocol with injection on E14 compared to injection on E17.5 in our study. Together, the previous and our present findings emphasize the importance of IOF timing and formulation of injected solution.

The higher bioavailability of creatine monohydrate used in our study may explain the rapid elevation of IGF1 and MYOG gene levels and consequently acceleration of muscle cell differentiation. Indeed, results from Deldicque et al. (2007b) and Fernyhough et al. (2010) point toward the role of creatine in accelerating the differentiation rate of myogenic cell lines *in vitro*. This is achieved by activating the p38 and the ERK1/2 mitogen-activated protein kinase (MAPK) as well as the phosphatidylinositol 3-kinase (PI3K) pathways that are known to be key signaling cascades in the differentiation process of myoblasts (Gredinger et al., 1998; Zester et al., 1999; Li, 2000; Li and johnson, 2006). In addition, a previous study showed an induction of IGF1 levels and higher breast muscle percentage in response to dietary guanidinoacetate―a creatine precursor―in broilers (Michiels et al., 2012). IGF1 and MYOG, were shown to be involved in myoblast proliferation, terminal differentiation, and muscle hypertrophy in broilers (Halevy et al., 2001; Halevy et al., 2004). An opposing trend is shown for MyoD expression with the lowest gene expression levels found in the IOF creatine group at hatch. MyoD has a key role in myogenic commitment and lineage of muscle progenitors and is also involved in the proliferation of satellite cells which serve as a pool for newly synthesized myotubes post hatch (Relaix et al., 2004; Gros et al., 2005; Kassar-Duchossoy et al., 2005). Overall, it is plausible that the energy boost achieved by IOF with creatine monohydrate has accelerated the differentiation of myogenic cells while slowing down their proliferation, resulting in premature differentiation with smaller size myofibers on day 14 post-hatch. These findings could be speculated as early signs for developmental abnormalities in the breast muscle (Halevy and Velleman, 2022). Additional analyses such as for collagen and fat accumulation as well as overall morphometry at later time points will shed more light on this speculation.

**FIGURE 5:**
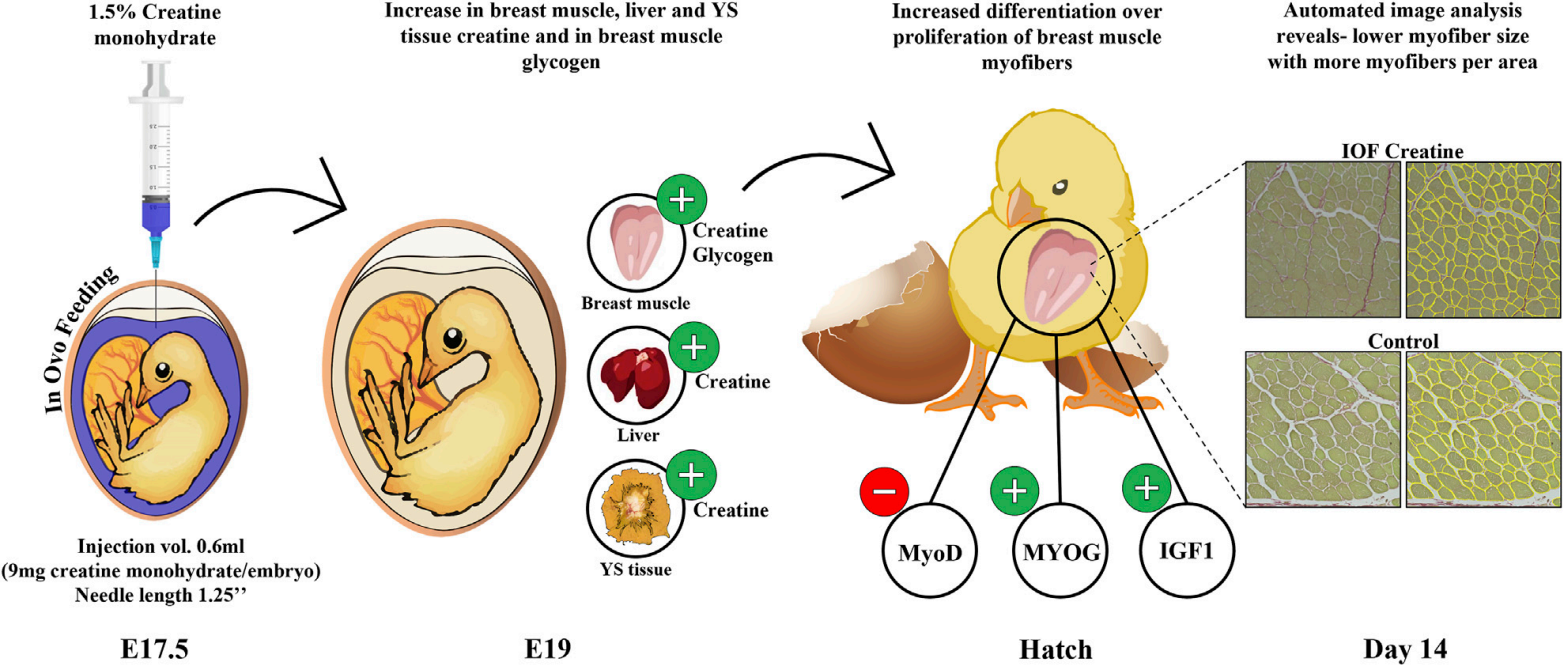
Graphical illustration demonstrating the effect of IOF with creatine monohydrate on energy resources (creatine and glycogen) of chicken embryos and the mode of effect on muscle growth during the pre-to post-hatch period. Our study shows an increase in creatine levels in breast muscle, liver, and YS tissue and in glycogen levels in breast muscle towards hatch. This in turn may influence gene expression changes in the IOF creatine group at hatch with modulatory effects on muscle development through differentiation over-proliferation of breast muscle cells. This is shown in reduced MyoD expression over increased MYOG and IGF1 expression and in reduced myofiber size in IOF creatine group compared to control.

In summary, our study provides important insights into the optimal procedure for IOF (Figure 1). Moreover, as seen in Figure 5, this study demonstrates that in-ovo creatine monohydrate enrichment of late-term embryos promotes their energy levels towards hatch and modulates early post-hatch muscle development through altered expression of myogenic-related genes. This is implied by higher expression levels of IGF1 and MYOG genes, related to differentiation of myogenic cells, and lower expression of MyoD, related to their proliferation. Thus, resulting in a significantly higher number of myofibers per area with smaller size on day 14 post-hatch. Altogether, the scope of this study exhibits a short-term potential of IOF with highly bioavailable creatine molecule in modulating post-hatch muscle growth and development. Therefore, it is important to examine the long-term effects as well as the formulation of IOF for its potential applications to overcome the current challenges related to muscle development and abnormalities in commercial broiler production.

## Data Availability

The original contributions presented in the study are included in the article/Supplementary Material, further inquiries can be directed to the corresponding author.
