# Supplementary material for: In-ovo feeding with creatine monohydrate: implications for chicken energy reserves and breast muscle development during the pre-post hatching period
Source: Front Physiol. 2023 Dec 14;14:1296342. doi: 10.3389/fphys.2023.1296342 (PMC10752974; doi:10.3389/fphys.2023.1296342)
Supplement: Supplementary file 1 [file DataSheet1.PDF]

In-ovo feeding with creatine monohydrate: implications for chicken energy reserves and breast muscle development during the pre-post hatching period

Jonathan Dayan[1], Tal Melkman-Zehavi[1], Noam Goldman[2], Francesca Soglia[3], Marco Zampiga[3], Massimiliano Petracci[3], Federico Sirri[3], Ulrike Braun[4], Vivienne Inhuber[4], Orna Halevy[1], Zehava Uni[1*]

[1]Department of Animal Science, The Robert H. Smith Faculty of Agriculture, Food, and Environment, The Hebrew University of Jerusalem, Rehovot 7610001, Israel
[2]Koret School of Veterinary Medicine, The Robert H. Smith Faculty of Agriculture, Food, and Environment, The Hebrew University of Jerusalem, Rehovot 7610001, Israel
[3]Department of Agricultural and Food Sciences, Alma Mater Studiorum - University of Bologna, Via del Florio, 2, 40064 Ozzano dell'Emilia/Piazza Goidanich, 60, 47521 Cesena, Italy.
[4]AlzChem Trostberg GmbH, Dr.-Albert-Frank-Straße 32, 83308 Trostberg, Germany

[*]Corresponding Author: Z., Uni. Department of Animal Science, The Robert H. Smith Faculty of Agriculture, Food, and Environment, The Hebrew University of Jerusalem, Rehovot 7610001, Israel
Email: zehava.uni@mail.huji.ac.il; Phone: +972-8-9489205; Cellular: +972-546999877

SUPPLEMENTARY TABLES

Supplementary Table 1. Creatine levels per tissue from E17 until Day 1 post-hatch in Control, IOF Creatine, and IOF NaCl groups

Day	Treatment	Dry weight concentration (mg/g)			Total amount in tissue (mg)		
		Breast muscle	Liver	YS tissue	Breast muscle	Liver	YS tissue
E17	-	8.28 ± 0.41	0.15 ± 0.02	0.13 ± 0.016	1.37 ± 0.15	0.023 ± 0.004	0.57 ± 0.06
E19	Control	9.12 ± 0.51	0.14 ± 0.02[b]	0.18 ± 0.05[b]	1.88 ± 0.1[b]	0.030 ± 0.004[b]	0.55 ± 0.16[b]
	IOF NaCl	3.02 ± 0.43	0.13 ± 0.02[b]	0.25 ± 0.04[b]	2.08 ± 0.13[b]	0.028 ± 0.006[b]	0.59 ± 0.04[b]
	IOF Creatine	10.29 ± 0.65	0.3 ± 0.05[a]	0.63 ± 0.016[a]	2.73 ± 0.11[a]	0.075 ± 0.013[a]	1.41 ± 0.17[a]
Hatch	Control	8.13 ± 0.64	0.13 ± 0.034	0.15 ± 0.02[b]	2.32 ± 0.24	0.05 ± 0.01	0.22 ± 0.04[b]
	IOF NaCl	6.71 ± 0.31	0.15 ± 0.03	0.16 ± 0.04[b]	2.21 ± 0.13	0.054 ± 0.01	0.21 ± 0.04[b]
	IOF Creatine	7.88 ± 0.79	0.21 ± 0.04	0.47 ± 0.04[a]	2.41 ± 0.26	0.075 ± 0.013	0.63 ± 0.11[a]
D1	Control	8.975 ± 0.62	0.054 ± 0.004	0.060 ± 0.008	3.59 ± 0.36	0.028 ± 0.003	0.060 ± 0.01
	IOF NaCl	8.902 ± 0.17	0.055 ± 0.005	0.047 ± 0.005	3.78 ± 0.14	0.030 ± 0.003	0.040 ± 0.004
	IOF Creatine	10.017 ± 0.53	0.059 ± 0.006	0.095 ± 0.033	4.07 ± 0.22	0.033 ± 0.003	0.086 ± 0.037

Creatine dry weight concentration (mg/g) and total tissue amount (mg) of breast muscle, liver, and yolk sac (YS) tissue. Lowercase letters denote cases significantly different between treatments at each time point, as derived from Tukey's HSD test ($P \leq 0.05$), n=6 per treatment and day.

Supplementary Table 2. Glycogen levels per tissue from E17 until Day 1 post-hatch in Control, IOF Creatine, and IOF NaCl groups

Day	Treatment	Dry weight concentration (mg/g)			Total amount in tissue (mg)		
		**Breast muscle**	**Liver**	**YS tissue**	**Breast muscle**	**Liver**	**YS tissue**
E17	-	16.00 ± 0.52	87.09 ± 7.52	24.24 ± 2.43	2.63 ± 0.22	14.03 ± 0.8	104.5 ± 8.92
E19	Control	17.74 ± 0.87	55.77 ± 9.66	23.89 ± 3.62	3.66 ± 0.16[b]	12.79 ± 2.76	69.92 ± 9.62
	IOF NaCl	15.35 ± 1.38	55.98 ± 6.26	27.76 ± 2.81	3.54 ± 0.34[b]	11.38 ± 1.62	69.96 ± 6.21
	IOF Creatine	18.32 ± 0.66	60.13 ± 9.08	34.19 ± 5.54	4.89 ± 0.19[a]	15.11 ± 2.4	75.57 ± 6.98
Hatch	Control	5.92 ± 1.85	9.76 ± 3.2	8.03 ± 2.004	1.74 ± 0.58	3.88 ± 1.33	12.41 ± 3.52
	IOF NaCl	6.25 ± 1.68	9.46 ± 3.09	10.06 ± 2.75	2.02 ± 0.56	3.82 ± 1.39	12.93 ± 3.3
	IOF Creatine	7.57 ± 1.45	16.29 ± 4.71	15.11 ± 3.78	2.44 ± 0.61	5.77 ± 1.62	19.55 ± 4.79
D1	Control	20.24 ± 2.61	141.96 ± 18.79	15.62 ± 3.79	8.01 ± 1.02	75.12 ± 12.16	12.83 ± 1.27
	IOF NaCl	20.58 ± 2.88	135.3 ± 19.8	19.24 ± 2.95	8.88 ± 1.49	76.49 ± 13.28	16.95 ± 2.27
	IOF Creatine	18.39 ± 1.26	139.42 ± 7.75	15.75 ± 2.07	7.51 ± 0.6	78.25 ± 4.84	11.74 ± 0.92

Glycogen dry weight concentration (mg/g) and total tissue amount (mg) of breast muscle, liver, and yolk sac (YS) tissue. Lowercase letters denote cases that are significantly different between treatments at each time point, as derived from Tukey's HSD test ($P \leq 0.05$), n=6 per treatment and day.

Supplementary Table 3. Body and tissue weights from E17 until Day 1 post-hatch in Control, IOF Creatine, and IOF NaCl groups

Day	Treatment	Body weight (g)	Breast muscle weight (g)	% Breast muscle of body weight	Liver weight (g)	YS tissue weight (g)
E17	-	43.93 ± 0.61	1.08 ± 0.04	2.46 ± 0.07	0.53 ± 0.02	13.52 ± 0.43
E19	Control	46.43 ± 0.73	1.21 ± 0.05	2.61 ± 0.15	0.64 ± 0.02	11.63 ± 0.43
	IOF NaCl	46.59 ± 2.44	1.29 ± 0.04	2.77 ± 0.14	0.61 ± 0.05	12.10 ± 1.78
	IOF Creatine	45.37 ± 0.61	1.31 ± 0.05	2.89 ± 0.12	0.68 ± 0.01	9.93 ± 0.55
Hatch	Control	42.58 ±1.49	1.39 ± 0.09	3.26 ± 0.12	1.09 ± 0.04	4.28 ± 0.21
	IOF NaCl	44.29 ±1.17	1.61 ± 0.09	3.64 ± 0.15	1.08 ± 0.05	4.57 ± 0.35
	IOF Creatine	44.50 ±1.70	1.55 ± 0.15	3.48 ± 0.22	0.97 ± 0.02	5.37 ± 0.51
D1	Control	50.05 ± 1.50	2.04 ± 0.13	4.04 ± 0.20	1.48 ± 0.07	2.66 ± 0.38
	IOF NaCl	51.20 ± 1.61	2.21 ± 0.08	4.32 ± 0.11	1.59 ± 0.07	2.95 ± 0.30
	IOF Creatine	51.62 ± 1.60	2.22 ± 0.05	4.30 ± 0.15	1.63 ± 0.04	2.53 ± 0.37
D3	Control	85.28 ± 1.93	5.27 ± 0.31	6.18 ± 0.39	-	-
	IOF NaCl	84.76 ± 1.96	5.22 ± 0.39	6.16 ± 0.36	-	-
	IOF Creatine	79.69 ± 2.92	5.48 ± 0.29	6.88 ± 0.22	-	-
D6	Control	156.90 ± 7.16	13.76 ± 0.63	8.70 ± 0.13	-	-
	IOF NaCl	152.30 ± 4.41	14.10 ± 0.87	9.26 ± 0.32	-	-
	IOF Creatine	157.17 ± 5.03	14.73 ± 0.34	9.37 ± 0.34	-	-
D14	Control	598.14 ± 10.62	98.37± 2.91	16.44 ± 0.29	-	-
	IOF NaCl	580.92 ± 14.12	95.79 ± 3.76	16.49 ± 0.47	-	-
	IOF Creatine	579.38 ± 9.21	93.46 ± 4.42	16.13 ± 0.57	-	-

Data of body weight, liver weight, yolk sac (YS) tissue weight and breast muscle weight and relative weight (%). As denoted from Tukey's HSD test ($P \leq 0.05$), no significant differences were found between groups, n=6 per treatment and day.
